# Mental Excitement

**Published:** 1889-03-30

**Authors:** 


### THE DOCTOR'S PULPIT.

### MENTAL EXCITEMENT.

THE Quakers are a calm people, and usually live long. They would probably live longer still but for their intermarriages. On the other hand, ardent politicians are often the subjects of tremendous excitement, and yet they too, as a class, manage to sustain life to a good old age. It would appear, then, that calmness of temperament has some advantages, but that excitement of itself is not necessarily injurious. The object of this paper is not to denounce excitement and to laud calm, but to persuade people to think out for themselves what is the best temper and habit of mind for health and happiness.

In the main, an ordered sobriety of behaviour is the condition most favourable to health; just as calm weather favours the growth and blooming of plants or the prosperous voyaging of a ship at sea. But plants are not injured by an occasional gale, provided it is not too fierce. They will even endure and profit by a storm if it be not of such severity as to break them down or tear them up by the roots. It is said that the brave oak in a hurricane clings with added tenacity to the soil, and after each successive attack of its enemy, spreads its roots both wider and deeper, as if to say, "I am here to live a thousand years, and I will stand my time in spite of all the fury you can bring to bear upon me." Once in a way, however, the storm is irresistible, and the monarch of the forest lays his proud head in the ground.

A certain eminent politician is famous for having usually three main points to his speech; and old-fashioned divines of the evangelical type would have considered it dangerously near heresy to have cast a sermon into any other mould than that of three heads and an application. We have, therefore, a not undignified sanction for dividing our thoughts here into three principal parts. Following these lines, we point out that mental excitement may be of three kinds—harmless, profitable, and injurious.

It is possible that some ladies find a mild kind of excitement in meeting together by the half-dozen for afternoon tea. The fragrant herb is undoubtedly a stimulant; and when properly brewed and taken hot, with a judicious admixture of cream, it generally has the effect of loosening the strings of the tongue. Under its quickening and expansive influence it is possible for ladies to take an eager and pleasurable interest in all their neighbour's most private concerns, and to discover and discuss a variety of circumstances connected with him which to duller men appear so microscopic and insignificant as to deserve no attention at all. This form of excitement may usually be described as absolutely harmless. It would be both inappreciative and unkind to hint that it might possibly be silly.

One must suppose that the average mortal experiences some kind of roused feeling in listening to an ordinary sermon. If not, why is there such a preparation of hats and bonnets, gloves and boots, with other accessories, every Sunday morning? Your ordinary Englishman would not miss his Sunday's sermon for the world. Yet if it is a stimulus, it is usually of a type of mildness which it would be altogether impossible to exceed. That it frequently affects the heart of the hearer hardly anyone would assert; that it touches his head in the smallest degree still fewer would venture to affirm. And yet there must be some faintest flutter somewhere in the consciousness, or surely people would stay at home and exclude Sunday bonnets from their milliners' bills. If there be excitement it is, as we have said, of a type whose delicate innocence cannot be surpassed, and can do no harm to man, woman, or long-coated baby.

Profitable excitement is quite common, and may be illustrated from almost every department of life in this country. Two rival schools playing at football, with the margins of the field lined by the friends of both parties, soon beget a fine storm of opposed enthusiasms. Not long ago the writer witnessed the international match at Edinburgh. English and Scotch were alike determined to win. The very air seemed to share in the mood of the hour. Most of the onlookers were Scotch. How they shouted, how they yelled; how they held their breath with immeasurable suspense for a moment, and then burst out again with an enthusiasm as uncontrollable as the rushing of the maddened wind. "Go it Scotland!" resounded from a thousand throats with an intensity of energy sufficient of itself to vanquish any enemy with mere astonishment, except, of course, an Englishman. One university professor, who will remember the occasion if he should read this paper, was literally carried into a new world—a world made up of Jacobitism, national pride, "Scots wha hae," schoolboy memories, and a thousand sentiments of the like kind. No excitement possible to men could have been greater. Was it healthy? Undoubtedly! Most of the people who witnessed the contest were exalted, expanded, vivified, and transformed both in body and mind for a brief and triumphant hour. Nothing but good results were likely to follow that excitement. Healthy play, innocent amusements, music, dancing, a dinner-party, a brisk gallop, a political meeting, and a really good sermon—all these rouse the mind, excite the circulation both in the brain and throughout the body, promote physiological activity and render great and incalculable service. Occasional, nay more or less regular excitement thus produced is better for health and happiness than the studied and unnatural sobriety practised by the Quaker. The man who cannot yield himself up entirely to these simple delights when they come in his way, is in a decidedly abnormal and decidedly unhealthy condition—a condition in which he would do well to seek competent medical advice.

Unhealthy excitement is the third kind we have to consider. The most common form of this is uncontrollable rage. In olden times, when men lived much in the open air, ate and drank like pigs, and slept like the same unclean brutes, a fit of savage passion was of common occurrence, and did not leave very serious consequences behind. But in these days, when the nervous system is a thousand times more sensitive and delicate, when digestion is doubtful, and sleep a coy goddess, easy to drive away and difficult to bring back again, a man should think twice before he allows himself to cross the border line between reined-in anger and furious, runaway rage. Such a state of mind, now euphemistically called a "nerve storm," takes it out of some men terribly. They feel after it a profound exhaustion and lassitude which may endure for hours. The digestion is often impaired, the liver disordered, and the heart and brain injured. A man who habitually indulges in violent rage is laying up for himself a store of ills against advancing age, the least of which may be a general palsy, the greatest a sudden and fatal fit of apoplexy.

Illicit passion is a highly dangerous form of excitement; drunkenness another; gambling another. All these are widely prevalent at the present time. The unlimited indulgence of unlicensed passion does not merely ruin the character; it plays rapid and fatal havoc with the constitution. It would be easy to give cases innumerable of strong and healthy young men brought down in a few years from perfect vigour and wholesomeness of body to conditions of loathsome and disabling disease, in many cases difficult or impossible of perfect cure, and in not a few resulting in speedy death. Medical men so frequently meet with these cases that their susceptibilities are apt to become dulled; but if any physician in large metropolitan practice would note all the cases of this kind which come under his care in ten years the results would be startling. In the case of some few whose specialty lies in this direction a record of all the work of their lifetime would be nothing less than a series of most terrible tragedies.

The excitement of gambling is by no means peculiar to the gaming table. There are streets in the City of London which at certain hours present the appearance of Pandemonium opened. Well-dressed men, with shiny hats stuck on their heads at an infinite variety of angles, crowd the streets and the footways, elbow one another, jostle the passengers, and bawl and bellow like so many costermongers at a donkey race. They are for the moment money-mad. Will they win or will they lose? And what is the figure which may indicate their safety or their danger? This is not legitimate work. It is gambling of the most exciting kind. Its consequences are evident enough in many cases. Characters once simple and innocent have been thrown to the winds. Coarseness and vulgarity are stamped upon the face. To the observant eye of the physician there are often evident incipient madness and premature old age. These are the kinds of excitement that undermine the foundations of conscience, wreck the morals, blast the fame, destroy the health, and scatter broadcast the seeds of disease and death. These and all other kinds of dangerous excitement a man who desires to be honest, healthy, and happy will shun as he would shun a pestilence. Their ways take hold on death; their gates are the gates of hell.

